# Strategies and limitations for fluorescence detection of XAFS at high flux beamlines

**DOI:** 10.1107/S1600577515001320

**Published:** 2015-02-17

**Authors:** Steve M. Heald

**Affiliations:** a X-ray Science Division, Advanced Photon Source, Argonne, IL 60439, USA

**Keywords:** XAFS detectors, fluorescence, detection strategies

## Abstract

The issue of detecting the XAFS signal from dilute samples is discussed in detail with the aim of making best use of high flux beamlines that provide up to 10^13^ photons s^−1^. Various detection methods are compared, including filters with slits, solid state detectors, crystal analyzers and combinations of these. These comparisons rely on simulations that use experimentally determined parameters. It is found that inelastic scattering places a fundamental limit on detection, and that it is important to take proper account of the polarization dependence of the signals. The combination of a filter–slit system with a solid state detector is a promising approach. With an optimized system good performance can be obtained even if the total count rate is limited to 10^7^ Hz. Detection schemes with better energy resolution can help at the largest dilutions if their collection efficiency and count rate limits can be improved.

## Abstract 2

Fluorescence detector strategies are compared for dilute XAFS samples on high flux beamlines.

## 1. Introduction   

One of the big advantages of the X-ray absorption fine structure (XAFS) method is the ability to obtain detailed chemical and structural information about very dilute components of a sample. This is enabled by fluorescence detection (Jaklevic *et al.*, 1977 ▸; Cramer *et al.*, 1988 ▸). In the dilute limit the fluorescence intensity is proportional to the absorption. The fluorescence lines for an element have unique characteristic energies, allowing direct detection of the component of the absorption due to a specific element. Thus, detection of the XAFS from elements approaching p.p.m. level dilution becomes possible. What is needed is a detector system that can isolate the fluorescence of the element of interest. As synchrotron radiation beamlines improve, it might be expected that the detection limits can be similarly improved. However, this can only happen if the fluorescence detectors also improve. In this paper we compare various detection strategies that can be employed at recently developed high flux XAFS beamlines. This extends upon calculations begun by Warburton (1986 ▸) in the light of modern detector technology and beamline optics, using results from measurements of actual detector performance. While the emphasis is on XAFS applications that typically require good signal to noise, many of the same considerations apply to other applications of X-ray fluorescence such as X-ray fluorescence microprobes for imaging.

Currently three types of detectors are used: filter–slit systems, multi-element solid state detectors and crystal analyzers. Sometimes these schemes can be combined. The primary goal is to separate the fluorescence line(s) of interest from the background. The background can be due to elastic or inelastic scattering, or fluorescence from other elements in the sample. For example, the cross section for scattering is typically about 1–3% of the absorption cross section. Thus, the background can be very significant when the element of interest reaches the p.p.m. level. Polarization can be used to minimize the scattering, but this becomes less effective when the goal is to collect a large solid angle.

The three detector types take different approaches. For the filter–slit system (Stern & Heald, 1979 ▸) it is easy to maximize the collection solid angle to maximize the fluorescence signal, but it only reduces the background by using a low-pass filter. This can work well when the background is due to scattering, but not so well if there are interfering fluorescence lines. While it can be combined with energy-resolving detectors, this is not required and the count rate when used with integrating detectors is essentially unlimited.

Solid state detectors, typically Si or Ge, have energy resolution good enough to separate the fluorescence from most of the background. Their main limitation is a maximum counting rate of about 10^5^–10^6^. When the background becomes large, the fluorescence signal will be limited by the need to restrict the solid angle to avoid saturation. Systems with multiple detectors (up to several hundred) have been developed to mitigate this issue, but a high flux beam can still overwhelm these detectors. Also, as will be shown later, the scattering background can leak into the fluorescence channel, giving an ultimate limit to the performance. However, solid state detectors can be easily combined with the other detector systems that remove some of the background before it reaches the detector.

For the ultimate in energy resolution, diffraction-based crystal analyzers have been developed. There are basically two types: (i) focusing systems such as Johann and Johansson cut crystals in the Rowland circle geometry (Hastings *et al.*, 1979 ▸; Marcus *et al.*, 1980 ▸; Bergmann & Cramer, 1998 ▸; Sutton *et al.*, 1994 ▸; Welter *et al.*, 2005 ▸) or (ii) non-focusing analyzers such as those based on log-spiral crystals (Zhong *et al.*, 1999 ▸; Kropf *et al.*, 2003 ▸, 2005 ▸; Pease *et al.*, 2000 ▸), arrays of flat crystals (Mattern *et al.*, 2012 ▸; Dickinson *et al.*, 2008 ▸) or multilayers (Zhang *et al.*, 1999 ▸). There has also been use of polycapillary coupling optics to enhance the collection efficiency of crystal analyzers (Heald *et al.*, 2012 ▸; Kirkland *et al.*, 1995 ▸; Szlachetko *et al.*, 2010 ▸). The focusing systems can have excellent background suppression, but generally have limited collection efficiency. Non-focusing arrangements are much better at collecting large solid angles, but because large area detectors are needed they can allow more background to leak through.

In this paper, both experiment and analysis is used to compare the various detector methods, with the aim of guiding future development to take best advantage of high flux beams. It starts with a consideration of the signals available and ultimate performance possible with an ‘ideal’ detector.

## 2. Signals available   

### 2.1. Signal-to-noise considerations   

When background is present it is convenient to compare detectors by their effective counting rate, *N*_e_. This is the number of counts that would provide the same signal to noise in the absence of the background. There are two possible definitions for *N*_e_. Consider the total signal *N*_t_ = *N*_f_ + *N*_b_, where *N*_f_ is the fluorescence and *N*_b_ is the background. If the goal is to estimate the absolute value of *N*_f_ at a single incident energy then the relevant uncertainty is that for *N*_t_ − *N*_b_. If the uncertainty is dominated by counting statistics and not some systematic factor that affects both *N*_f_ and *N*_b_, then the uncertainty is 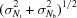 = 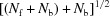 = 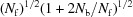. This is 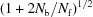 times larger than the noise for the fluorescence signal with no background. Thus, the effective counting rate with the same signal to noise is
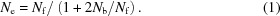
However, for XAS measurements we are more interested in the changes in the signal as the incident energy is changed. Any background removal is done by fitting smooth curves for a large number of points collected at different energies. In this case the more relevant noise is that for *N*_t_ alone, and the effective counting rate becomes
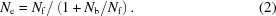
This definition will be used to compare detectors. As a consequence, when background is present the counting time to achieve a given signal to noise increases by 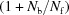. Thus, a good detector will maximize *N*_e_ by maximizing the collection efficiency for *N*_f_ and have the maximum discrimination of the background to reduce *N*_b_.

Detection of EXAFS or XANES requires better signal to noise than for simply detecting the presence of an element. At a minimum, a XANES measurement requires about 100 points each with 10^4^ or more effective counts, while for EXAFS the number of points is at least 200 with an average of 10^6^ or more effective counts. This means a whole spectrum would have about 10^6^ counts for XANES and 2 × 10^8^ counts for EXAFS. In both cases, high quality data would require five to ten times more counts. Thus, for a whole spectrum a goal is to collect about 10^7^ total effective counts for XANES and 10^9^ for EXAFS.

### 2.2. Examples for Cu   

The available signals from existing beamlines can allow detection to very high dilution if efficient detectors can be achieved. This can be illustrated by considering some example situations. Assume that the beamline supplies 10^13^ photons s^−1^, and the sample is Cu in a moderately absorbing matrix such as SiO_2_. If the Cu concentration is 10^−9^ at% (1 p.p.b.), the Cu in a thick sample would absorb about 1.1 × 10^5^ photons s^−1^. The Cu fluorescence yield is about 0.45. Considering the need for the fluorescence to escape, the realistic solid angle available is about 20% of the total for a flat sample mounted at 45°. Some of the fluorescence photons will also be reabsorbed before they can exit the sample. This will further reduce the signal by approximately 3. Thus, an ideal detector would detect about 3.2 × 10^3^ photons s^−1^, enough for XANES in about an hour. Similarly a 1 µmole solution of Cu in water would provide about 7.3 × 10^5^ photons s^−1^, enough for EXAFS measurements in about an hour. These types of concentration are not currently feasible. Current detectors are limited by some combination of the total counting rate, poor background rejection and low collection efficiency.

**Figure 1:**
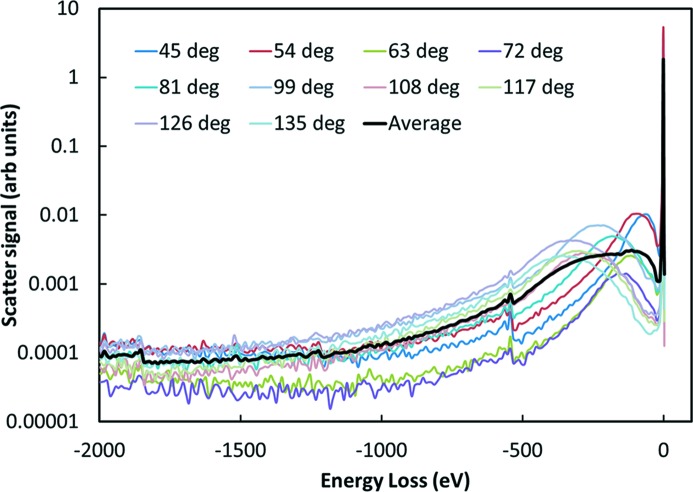
Measurement of the scattering spectra from a SiO_2_ sample with an energy resolution of about 1 eV. The indicated scattering angles were in the vertical direction, and the X-ray polarization was not a factor.

The above calculation is oversimplified since there will be sources of background at similar energies as the fluorescence lines. These cannot be rejected by the detector and will, thus, lower the effective counting rate. Fig. 1 ▸ shows a plot of the scattering from a SiO_2_ sample measured with high resolution (1 eV) crystal analyzers. There is a sharp elastic line, a broad Compton peak, and background extending to large energy loss that includes the tail of the electron-momentum-broadened Compton profile, multiple Compton scattering and X-ray Raman signals from O (edge near −540 eV) and Si (edge near −1840 eV). The Compton background has been considered in detail by Sternemann *et al.* (2008 ▸). These measurements were made in the vertical scattering geometry. For the more typical horizontal detection case the horizontal polarization of the synchrotron can be used to reduce the scattering. However, most of the background signals have similar polarization dependence to the elastic scattering, and their relative contributions are largely independent of the detection geometry.

For our Cu example, the Cu fluorescence is about 900 eV below the Cu edge and to cover the entire *K*_α1_ and *K*_α2_ signals would require a bandwidth of about 30 eV. In this region the background is about 1 × 10^−4^ as strong as the elastic peak. Considering the bandwidth needed to collect all of the fluorescence, the total background is expected to be about 3 × 10^−3^ times the elastic scattering peak. Measurements were made using a large-area ion chamber of the scattering background from SiO_2_. Over a 52° horizontal and vertical angle (∼6% of 4π) and taking advantage of the polarization in the typical horizontal arrangement, the total scatter was measured to be about 6 × 10^−5^ of the incoming beam flux at 10 keV. This is a smaller solid angle than the examples, and the scatter fraction would be even larger for larger collection angles (see Appendix *A* [App appa] for more details on the polarization dependence). At these energies the primary contributor is the elastic peak. Combining this measurement with the results in Fig. 1 ▸ we can estimate the background in a 30 eV bandwidth to be at least 2 × 10^7^. In our examples, this would lower the effective counting rates to less than 1 Hz for the 1 p.p.b. sample and 2.6 × 10^4^ Hz for the 1 µmole sample. The 1 p.p.b. XANES becomes impractical and the 1 µmole EXAFS would be difficult (∼10 h counting time). There could be some improvement with a higher resolution detector that only collected the more intense *K*_α1_ emission line, but generally it is more difficult to collect a large solid angle as the energy resolution is improved. Also, note that, if an energy-dispersive detector is used to separate the fluorescence signal, based on the measurements above the total (scattering + fluorescence) signal it would need to handle is greater than 4 × 10^9^ if we attempt to collect the 20% solid angle.

These estimates illustrate some of the fundamental limits in detecting XAFS. At high dilution the effective counting rate drops as the concentration squared (Warburton, 1986 ▸). For the examples above it seems that about 30 p.p.b. for XANES and 1 µmole for EXAFS would be the practical limits. Thus, the goal is to develop detector systems that can handle these large signals and approach these limits.

## 3. Comparison of detectors   

In this section the characteristics of the three detector types are considered in more detail to look at the parameters that limit their performance. These characteristics are then used to compare their performance individually and in combination.

### 3.1. Solid state detectors   

Solid state detectors (SSDs) or more generally energy-dispersive detectors collect all of the photons and output a signal proportional to their energy. Thus, they need to handle the total count rate that is incident upon them. Currently the best detectors in terms of count rate and resolution are the silicon drift detectors (SDDs) (Strüder *et al.*, 1998 ▸; Woicik *et al.*, 2010 ▸). With proper dead-time correction they can handle about 10^6^ counts s^−1^ with approximately 3% energy resolution. Thus, an approximately 1000 element detector would seem to approach the performance needed. Such a detector seems possible considering that 400 element detectors have been developed using somewhat less capable technology, and efforts are underway to apply the same thing to SDD technology. However, entirely different technology will be required to reach the 30 eV (∼0.3%) energy resolution considered in §2.

**Figure 2:**
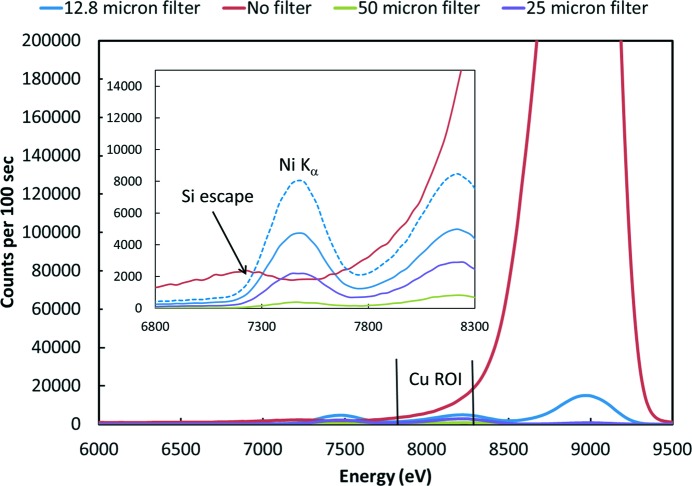
Measured spectra of the scattering from a SiO_2_ sample using a silicon-drift detector and Ni foil filters with the listed thicknesses. The inset shows details of the region near a typical region of interest (ROI) for the Cu *K*_α_ fluorescence. The dashed line is the 12.8 µm data corrected for the filter absorption below the edge.

The impact of the finite energy resolution is shown in Fig. 2 ▸. This is the scattering from a SiO_2_ sample as measured by a SDD. The inelastic tail of the scattering peak and the finite resolution of the detector results in about 1.6% of the scattered signal showing up in the Cu fluorescence channels when no filters are used. Improved energy resolution is possible with detectors based on superconductors (Day *et al.*, 2003 ▸; Irwin *et al.*, 1996 ▸), but the count rates are limited to hundreds of hertz. Work is underway to increase the pixel count of these detectors, but they are a long way from handling the count rates needed. They do seem possible candidates, however, for combined solutions as discussed in §4.

### 3.2. Crystal analysers   

There are several types of crystal analysers. Generally they are applied when superior energy resolution is needed. The measurements shown in Fig. 1 ▸ are typical of bent Si or Ge analysers operated in the Rowland circle geometry. When operated near backscattering they can provide excellent energy resolution, but typically a different crystal reflection would be needed for each fluorescence line. Also, the intrinsic resolution of the crystal reflection is much better than needed resulting in loss of signal. A typical case would have a 10 cm-diameter crystal located 1 m from the sample collecting about 0.06% of the total solid angle. The intrinsic resolution of typical Si or Ge reflections is 1 eV or less. The line widths of the emission lines vary with element, but generally are larger than the crystal acceptance. Also, the *K*_α1_ and *K*_α2_ lines are separated by 10 eV or more. Thus, perfect crystal analyzers are less than 100% efficient. Mosaic crystals such as high oriented pyrolitic graphite (HOPG) or damaged LiF can have much broader acceptances. However, this comes with a reduction in peak reflectivity. For example, the measured reflectivity (Heald *et al.*, 2012 ▸) for Ni *K*_α_ with HOPG is about 30%.

Due to the limitations of perfect crystal analyzers in the Rowland circle geometry, other designs have been considered. Early efforts used multiple flat or singly bent mosaic crystals to approximate the cylinder of revolution of the Rowland circle (Hastings *et al.*, 1979 ▸; Marcus *et al.*, 1980 ▸). These collected 1–2% of 4π with a diffraction efficiency of 10–30%. Thus, the net efficiency is still low, but since they are partially focusing the background rejection is good.

There have also been designs based on the non-focusing log-spiral crystal shape. This design allows a large collection angle but also requires a large area detector and, thus, it is difficult to completely eliminate the background. A detailed discussion for Laue geometry crystals can be found by Kropf *et al.* (2003 ▸). The strain in bent Laue crystals can be used to optimize the energy resolution with some loss in efficiency. Typically these crystals give 10–30% diffraction efficiency, and have collection area of ∼1%. To date the largest solid angle crystal detector is an HOPG analyser deposited on a log-spiral of revolution in Bragg geometry (Pease *et al.*, 2000 ▸). It collects 17% of 4π with an efficiency estimated to be 20%. However, the energy resolution of this detector is only about 4%, similar to a solid state detector. Also, it is optimized for a small energy range. Its main advantage is the ability to bypass the count rate limitations of solid state detectors when there are strong interfering fluorescence lines from concentrated components of the sample.

There is one additional factor to consider for crystal detectors. To maintain energy resolution they typically require a small focused beam. Beam sizes of ∼1 mm are often available at modern high flux beamlines. However, to achieve large collection angles with reasonable size crystals it is necessary to have short sample-to-crystal distances. This can result in the need for sub-100 µm beam size and some loss of incident flux. Also, for compact crystal arrangements the depth of view can be similar to the beam size. This means that for low absorption samples such as metalloproteins with millimeter or more of beam penetration the fluorescence might be efficiently captured only for the near-surface region.

### 3.3. Filter-based detectors   

Since the fluorescence is lower in energy than most of the scattering, an X-ray filter with an edge in between can be used to reduce the scattered background. For *K*-edge filters the absorption coefficient for the scattered radiation can be six to seven times higher than for the fluorescence signals. Since the transmission depends exponentially on the absorption coefficient, dramatic background reductions can be achieved with a small loss in the fluorescence signal. Also, since the filter is providing the energy discrimination a large-area non-energy-resolving detector can be used that can collect a very large solid angle. The main difficulty with this approach is minimizing the refluorescence from the filter that reaches the detector. This can be done with appropriate collimators (Stern & Heald, 1979 ▸). Given their simplicity and large collection efficiency, these detectors have been very popular and successful for samples with modest backgrounds.

**Figure 3:**
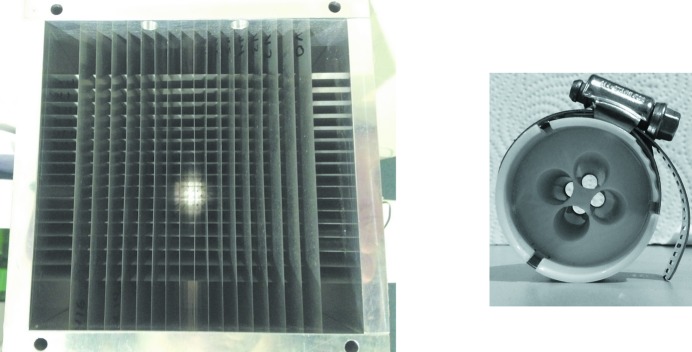
Photographs of the two types of collimators tested. Left – 2D collimator: a 2D grid of metal vanes designed for a large-area detector such as an ion chamber. The active region is 10 cm × 10 cm. Right – plastic collimator: a 3D printed plastic collimator designed for a four-element silicon drift detector.

Fig. 2 ▸ shows some examples of the background reduction that can be achieved with filters. These measurements were made using a SDD detector to look at what makes it past the filter. Two types of collimators were tested. These are shown in Fig. 3 ▸. The first, labeled 2D collimator, is a two dimensional grid of thin metal plates all aimed at the sample position. It is designed to be used with a large area detector such as an ion chamber. The second, labeled plastic collimator, was formed using rapid prototyping methods specifically for a four-element SDD detector. It consists of four truncated conical holes leading from each detector element to the sample position. The holes are truncated to be 20 mm-long and the sample is a total of 45 mm from the detector elements. Fig. 2 ▸ shows results for the plastic collimator. The 2D collimator was similar with three times better suppression of the filter fluorescence.

Fig. 2 ▸ shows the filters behaving as expected. The scattered signal is dramatically reduced and a small amount of Ni fluorescence leaks through the slits. By looking at the Ni *K*_α_ signal we can estimate the rejection ratio of the filter–slit system. For a non-energy-resolving detector this is defined as the Ni fluorescence (Ni *K*_α_ and *K*_β_) reaching the detector divided by the reduction in the scattering signal. The rejection depends somewhat on the filter thickness since for a thicker filter more of the Ni fluorescence is absorbed in the filter. For the 12.8 µm case the rejection ratio is determined to be 0.0075. If an energy-resolving detector is used we only need to consider the fraction of the Ni fluorescence that is within the Cu *K*_α_ region of interest (ROI). For typical settings this is only about 60% of the Ni *K*_β_ line. Since this line is about 15% of the intensity of the *K*_α_ line, the rejection ratio when used with a SDD detector would be 0.00059. Even this simple collimator provides very good rejection of the Ni fluorescence.

The dashed line in Fig. 2 ▸ shows the 12.8 µm data corrected for the filter absorption. This can be used to estimate the different contributions to the Cu ROI background. Aside from the filter fluorescence contribution, these are expected to include the intrinsic background from inelastic scattering and the tail of the large elastic scattering peak due to the finite resolution of the detector. The elastic scattering peak for the filter case is small, and we expect the background for that case to be dominated by the intrinsic background reduced by the below-the-edge Ni absorption. Since the dashed line does not match the no-filter case, the difference can be used to estimate the contribution of the elastic peak tail. This turns out to be about 0.005 of the elastic peak intensity, and the intrinsic background is about 0.011 of the elastic peak intensity. These numbers are used in some of the calculations to follow.

### 3.4. Comparison calculations   

**Figure 4:**
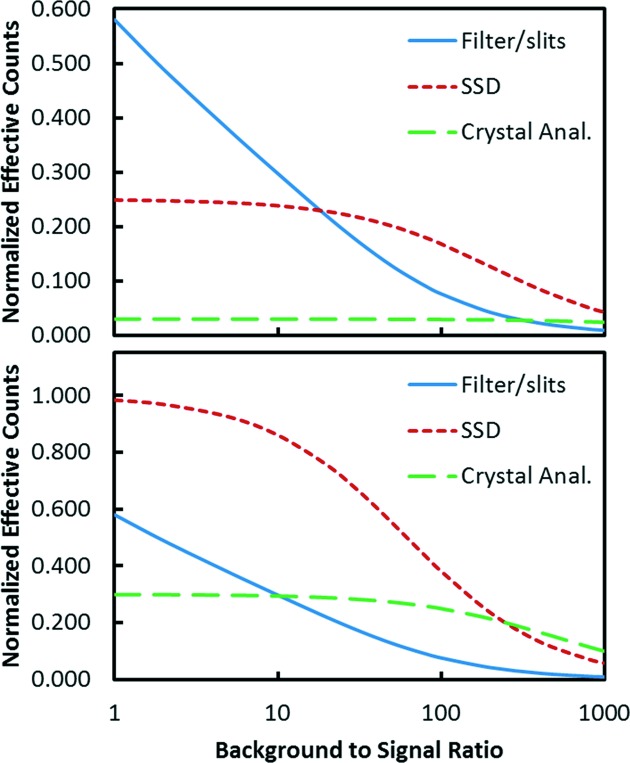
Comparison of the performance of the three detector types. The effective count rates are normalized to the total fluorescence signal available in the full solid angle. The bottom comparison is for detectors with equal solid angles. The top plot assumes that the solid state detector has a quarter of the solid angle and the crystal analyser solid angle is one tenth. In both plots the crystal detection efficiency is 30%.

Now that we have some basic detector properties, it is possible to make realistic calculations comparing their performance under various assumptions. Some examples are shown in Fig. 4 ▸ for a couple of cases as a function of the ratio of the scattering to the fluorescence for the raw signal. Details of the calculation methods are given in Appendix *A* [App appa]. These types of comparisons depend critically on the assumptions used. In Fig. 4 ▸ it is assumed that there are no count rate limitations. For the filter–slit system the parameters were those measured for the 2D collimator assuming a large-area ion chamber as a detector. The filters were assumed optimized as described in Appendix *A* [App appa], and the absorption parameters were characteristic of a metal *K*-edge foil filter. For the solid state detector the principal variable is the leakage of the scattering peak into the ROI for the fluorescence line. As discussed, there are two contributions to this: the intrinsic inelastic scattering background and the leakage of the scattering peak due to the tails of the resolution function. Both depend on the detector resolution. The first depends on the width of the ROI needed to collect the fluorescence and the second on the width of the scattering peak. From the data in Fig. 3 ▸ the sum of these contributions was taken as 1.6% of the scattering peak, characteristic of a SDD operated at 0.5 µs peaking time. For the crystal case there is more variation in potential parameters. For these calculations a somewhat idealized case was assumed. The only background was from inelastic scattering in a 30 eV bandwidth (2 × 10^−3^ of the scattered peak). Also, it was assumed this could be accomplished with 30% diffraction efficiency. In reality some of the additional background can be expected to leak into the detector, and as discussed it is difficult to combine large solid angles and high diffraction efficiency with good background rejection.

At the bottom of Fig. 4 ▸ it is assumed that all of the detectors collect the same large solid angle of 18% of 4π (90° vertical and horizontal collection angles). The results are plotted in terms of the background-to-signal ratio that would be seen by a non-energy-resolving detector such as an ion chamber. This is directly related to the concentration with a proportionality constant that depends on the details of the system. In this case the SSD is clearly superior except at the highest dilutions where the superior background rejection of the idealized crystal detector takes over. At the top of Fig. 4 ▸ somewhat more realistic solid angles are assumed. The SSD solid angle is reduced by 0.25 (4.5% of 4π) and the crystal is reduced by 0.1 (1.8% of 4π). When the solid angles are reduced the background-to-signal ratio can be reduced by taking advantage of the beam polarization since the scattering varies as 1 − cos^2^(θ) where θ is the angle away from the polarization direction. This was taken into account by numerically integrating this factor over a square detector face as described in Appendix *A* [App appa]. However, for plotting the results we continue to use the peak-to-background ratio of the full solid angle as a proxy for the concentration. This plot indicates why the filter–slit system has been a popular choice for moderately dilute samples.

### 3.5. Combining filters with solid state detectors   

It is simple to combine filters with solid state detectors. They can simultaneously be used to reduce the background and to reduce the total count rate to manageable values. In this section a number of cases are compared.

**Figure 5:**
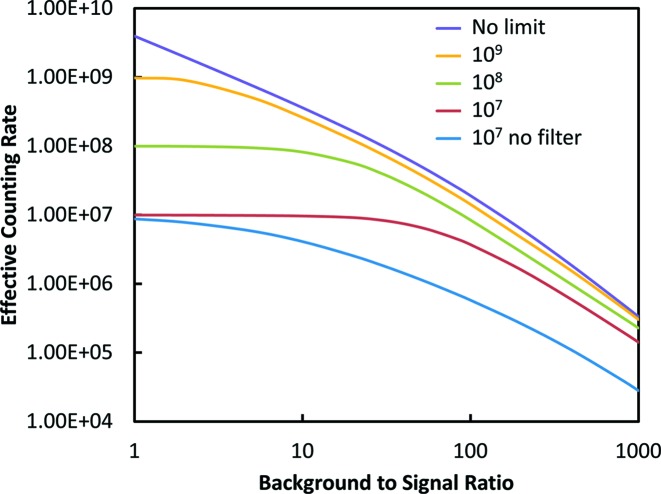
Effective counting rates for solid state detectors combined with an optimized filter–slit system for various count rate limits on the detector. For comparison the 10^7^ case is also shown without filters.

These results are summarized in Fig. 5 ▸. To generate these curves it is necessary to assume a value for the total scattering signal. For these examples the SiO_2_ value of 4 × 10^9^ for *I*_0_ = 10^13^ and a detector solid angle of 18% of 4π are used as a baseline for the background-to-signal ratio. This scattering combined with the background-to-signal ratio determines the fluorescence signal and the total counts in the detector. There are three ways to reduce the total counts to an acceptable level: reduction of the detector solid angle, addition of filters or reduction of *I*_0_. The first two are preferred since they reduce the background-to-signal ratio reaching the detector, improving the effective counting rate. For the optimization, the optimum combination of solid angle reduction and filters was determined to maximize the effective counting rate. As the solid angle is reduced, the reduction in the fluorescence and scattering signals were calculated as described in Appendix *A* [App appa]. The filter absorption is then applied reducing the background more than the fluorescence. The effective counting rate was then determined assuming the filter leakage parameters measured for the plastic collimator. Seeing as the solid angle goes to zero there is still some background from the required finite size of the detector and things like the imperfect polarization of the beam; the minimum solid angle allowed was 2% which corresponds to about 2% of the total scattering of the maximum solid angle. Similarly the maximum filter thickness was assumed to be μ*x* = 10 above the edge. If the total was still too high for the detector then *I*_0_ was reduced. These limits were only reached for low background-to-signal ratios where the effective counting rate was insensitive to these values.

Fig. 5 ▸ demonstrates a number of interesting observations. First, filters are of little help if the detector does not have any count rate limits. A SDD-type detector already has enough energy resolution to reject most of the background from a large scattering peak. Most of the background comes from inelastic scattering. However, when the count rate is limited, the curves tend to converge at large background-to-signal ratios. At high dilution the effective counting rate for the 10^7^ detector is only about a factor of two worse than the 10^9^ limit detector. Thus, if filters are used appropriately it is not necessary to push detectors to very high count rates.

There are also some interesting observations that can be made from the derived parameters. Some examples are shown in Table 1 ▸. As the detector count rate is limited, it is better to restrict the solid angle then increase the filter thickness. Also, it can be seen that when the sample becomes very dilute the effective count rate can actually be improved by restricting the solid angle. This occurs when the background detected in the fluorescence ROI becomes much larger than the signal even after the filtering.

### 3.6. Combining crystal analyzers with solid state detectors   

Since the results in §3.5 show that excellent results can be obtained even for restricted solid angles, crystal analyzers warrant further consideration. Existing designs can collect close to 2% of 4π, which is similar to the optimum for the lower count rate cases. Crystal analyzers also have the important property of rejecting possible lower energy fluorescence lines that could overwhelm the detector. The comparisons in §3.4 considered an idealized analyzer. Here we attempt to make some more realistic assumptions and combine them with solid state detectors. In real analyzers it is found that the background rejection is not perfect. Some of the scattering from the sample can scatter from the crystal, air paths or windows and make it into the detector. For a LiF-based barrel-style analyzer detector this was found to be about a 10^−4^ rejection ratio (Marcus *et al.*, 1980 ▸). For a bent Laue detector the leakage of U fluorescence into the Np fluorescence signal was found to be 0.003 (Kropf *et al.*, 2005 ▸). These lines are about 300 eV apart and presumably the rejection of the scattering would be better. However, both of these backgrounds can be minimized if a solid state detector is used to detect the analysed photons. To improve upon the filter/SSD combination it is important that the analyzer has better energy resolution than the SSD in order to reject more of the intrinsic background.

**Figure 6:**
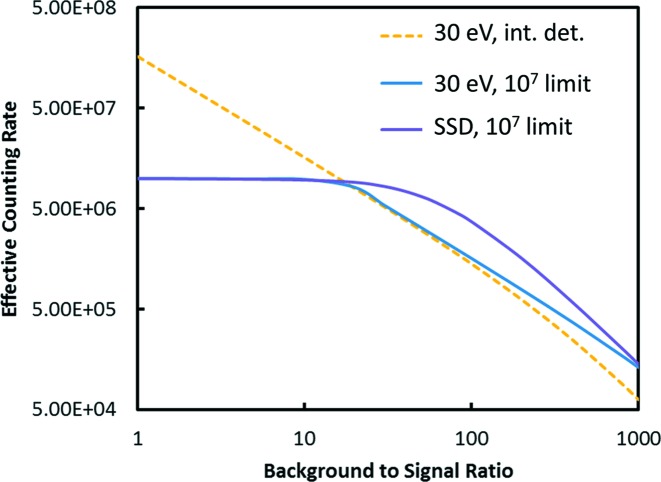
Calculations of the effective counting rates for a bent Laue-type crystal analyser with 30 eV resolution combined with an integrating detector or a solid state detector with 10^7^ count rate limit. Also plotted for comparison is the result for the same SSD combined with filters.

Fig. 6 ▸ shows some calculations for a crystal analyzer similar to the bent Laue case. The energy resolution was 30 eV, the scattering leakage was 0.003, the reflection efficiency was 0.3 and the solid angle 2%. In this case adding the solid state detector with 10^7^ count rate limit gives some improvement at large background ratios. However, a crystal analyzer with 10^−4^ scattering leakage would not really benefit from combination with a solid state detector. For comparison, the 10^7^ detector/filter combination is also shown. Except for the largest background ratios it is better due to the larger possible solid angle and greater detection efficiency.

## 4. Comparison with measurements   

**Figure 7:**
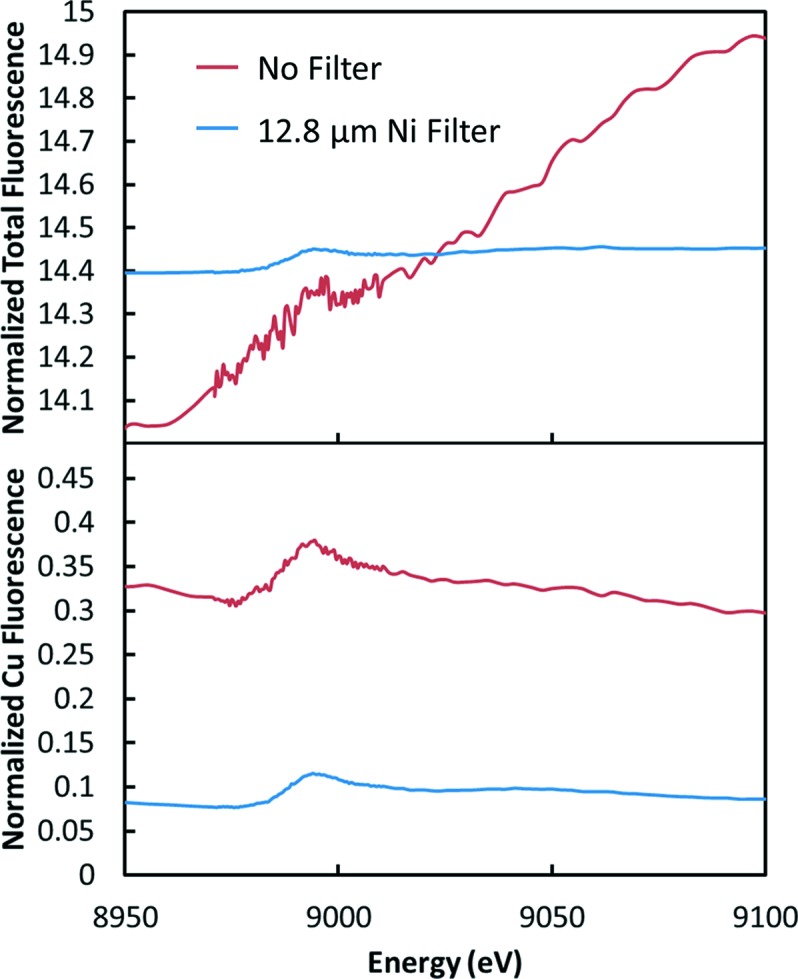
Fluorescence signals normalized to *I*_0_ as measured from a 55 µmole Cu solution using a four-element SDD detector and the plastic collimator. The top plot shows the total signal on the detectors summed over all energies, while the bottom is for the Cu ROI (7800–8280 eV). In the top panel the filter curve has been shifted upwards by 14 units vertically for comparison.

The calculations above are somewhat idealized since there might be sources of background that are not considered. To qualitatively validate the calculations, measurements were made on a series of dilute Cu solutions. The most dilute case was a 55 µmole CuCl_2_ solution contained in a 1.7 mm kapton tube. Fig. 7 ▸ shows the signals from this sample. These were made using the plastic collimator on a four-element Vortex detector with a detector-to-sample distance of 45 mm. Given the area of the detectors (170 mm^2^ total) this collected about 0.67% of 4π. Because of dead spaces between the detector elements, this is less than half of the collection area for a similar-size detector without dead space. The filter was a 12.8 µm Ni foil (μ*x* = 3.1 at 9000 eV). The top of Fig. 7 ▸ shows the data for the total counts on the detector. Note that the filter data have been shifted vertically. These data can be used to estimate the raw background-to-signal ratio. For the no-filter case it is difficult to see the edge. If we use the filter data and then remove the effect of the filter, the ratio is determined to be about 200. The bottom of Fig. 7 ▸ shows the same two scans, but looking at the data from the Cu ROI. Even for the no-filter case the detector does a decent job of removing the scattering background. However, it was necessary to attenuate the incoming beam by a factor of three to keep the total count rate near 100 K per detector element. Using the measured signal-to-backgrounds from Fig. 7 ▸ and the increase in count rate, the effective count rate for the filter case should be about 3.3 times better, which is in reasonable agreement with the data shown.

For the filter case the measured *I*_0_ was 4 × 10^11^ and the measured Cu signal edge step was 8000 Hz. With the background present (17000 Hz), this translates to an effective counting rate of 2600 Hz. If the incoming beam was increased to 1 × 10^13^, the effective counting rate with this solid angle and filter would be 65000 Hz. However, the total counts would be too high for the detector to handle. We would need a detector capable of about 10^7^. To test the calculations we can plug in the experimental values (background ratio of 200 over a similar solid angle and 3.1 filter thickness) into the model. We also need an estimate of the total scattering into the detector solid angle. This can be determined from the no-filter data. The calculated effective counting rate is then 1940 Hz resulting from a Cu signal of 6600 Hz and a background in the Cu ROI of 15800 Hz. The agreement is quite good considering the various differences in the calculation and experiment. The main difference is the sample geometry since a finite-thickness tube will allow better escape of the fluorescence. Thus the measurements provide validation that the presented comparisons are a reasonable approximation to reality.

The measured values can be used to calculate the signal for more dilute samples with higher flux and a detector that collects a larger solid angle. A detector with 10^7^ count rate limit, the optimum filter and *I*_0_ = 10^13^ would have an effective counting rate of 1.6 × 10^5^. For a sample ten times more dilute (5.5 µmole) the background will be the same and the fluorescence reduced by ten times, giving an effective count rate of 2200. If we do the same calculation for an ideal detector (ten times better resolution and no count rate limits), the effective count rate becomes 1.8 × 10^4^. This illustrates the advantage of better resolution to reduce the intrinsic background contribution.

Currently, superconducting detectors can provide the required 30 eV or better energy resolution at a few hundred Hz count rates. Efforts are underway to multiplex these (Eckart *et al.*, 2012 ▸). Potentially such a detector could have as many as a thousand elements giving a maximum total count rate of ∼2 × 10^5^ Hz. Using such a detector with an optimum filter on the 5.5 µmole example would give an effective count rate of 3700 Hz, already better than the 10^7^ limited SSD.

## 5. Discussion and conclusions   

As beamline performance is improved it is important to optimize detection strategies to make best use of available photons and to minimize radiation damage issues. A series of measurements and calculations were made comparing various detection methods for dilute XAFS experiments. Parameters typical of modern detectors were applied, but the conclusions are not very sensitive to the details. A key improvement over past comparisons is better consideration of the intrinsic inelastic scattering background, and a better treatment of the use of the X-ray polarization to suppress the background. The calculations have concentrated on the case where the background is dominated by scattering from the sample matrix. In this case it is clear that a high-count-rate solid state detector is the best choice. However, filters can significantly reduce the total count rate needed. Current multi-element detectors are beginning to achieve total count rates near 10^7^. This is sufficient to get most of the performance from a high flux beamline if combined with an appropriate filter–slit system. When the dilution is high, a 10^7^ rate-limited detector is reduced only by about a factor of two in performance compared with one with unlimited count rate. The calculations also illustrate the importance of using the polarization of the beam to reduce the scattering. Even for the high count rate detector, the effective count rate at large dilution was improved by restricting the solid angle. The fluorescence is approximately proportional to the solid angle while the scattering depends on a higher power. However, to gain this advantage it is important to restrict the scattering only to that originating from the sample. Here an appropriate slit system can play a role in reducing other scattering contributions.

The performance can also be improved by improving the energy resolution of the detectors. The inelastic scattering background will always have a contribution, but can be reduced by using a higher resolution detector. It appears that Si or Ge detectors are near their resolution limits. It is shown that high resolution superconducting detectors could become competitive for dilute samples at total count rates near 10^5^. Another approach to high resolution is the use of crystal analyzers. For the specific cases considered here they can become competitive at large dilutions if they can collect a few percent of 4π with reasonable efficiency. However, they can be used to great advantage when there are interfering fluorescence lines that cannot be eliminated by a filter. Also, with high resolution and good background suppression they can expand the useable solid angle range.

There is still room for improving detectors, both in total count rate and energy resolution. However, since the total count rate is already sufficient to obtain much of the available performance, improved energy resolution is likely to be most important.

## Figures and Tables

**Figure 8:**
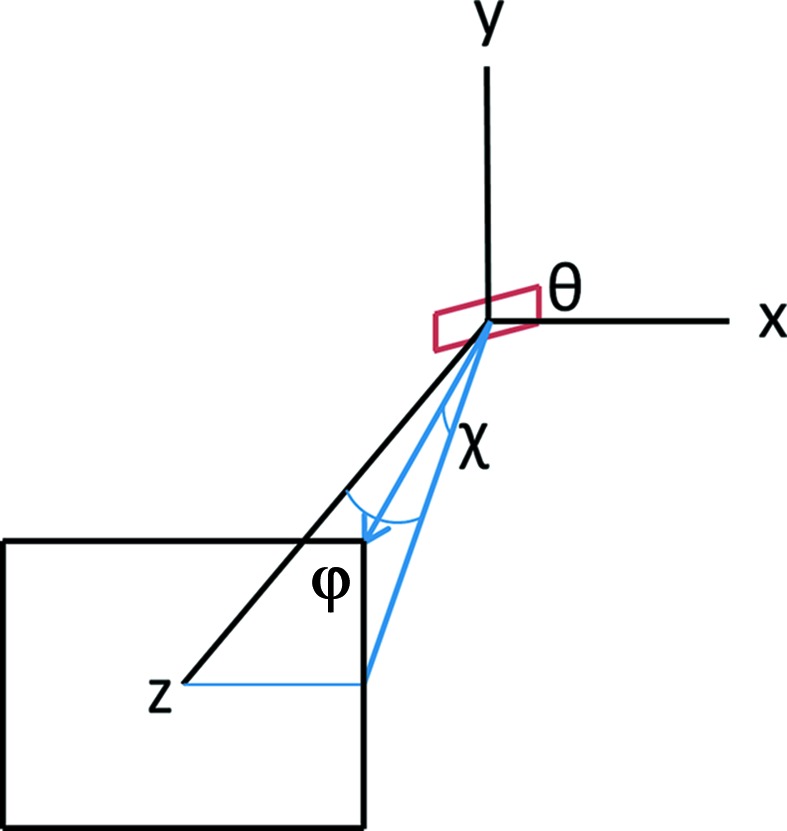
Geometry used in the calculations. θ is the angle the beam makes with the sample, and φ and χ are the angle to a point on the detector face. The detector is centered on the beam polarization direction that is along the *z* axis.

**Figure 9:**
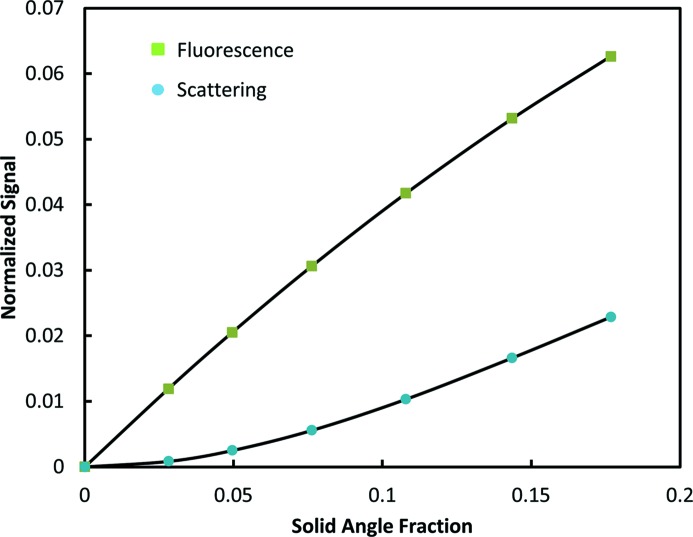
Angular dependence of the escape probability of the fluorescence and scattering for a thick sample. The solid angle is the fraction of 4π assuming a square detector face. The solid lines are polynomial fits to the calculated points.

**Table 1:** Values for the optimized filter absorption and solid angles for the best effective count rates when filters are combined with solid state detectors with the indicated count rate limits. The solid angles are the percentage of 4π and the filter values are the absorption coefficient just above the Cu edge (9000 eV)

	10^7^ count limit		10^8^ count limit		10^9^ count limit	
Background to signal	Solid angle	Filter	Solid angle	Filter	Solid angle	Filter
10	2.0	9.5	10.2	5.8	17.7	2.6
20	2.0	5.9	17.4	5.7	17.7	2.3
40	3.5	5.7	17.7	4.7	17.7	2.1
80	4.9	5.4	17.7	4.3	17.7	2.1
200	4.8	4.4	8.9	2.7	10.3	1.3
400	3.5	3.5	2.3	1.4	7.7	0.4
1000	2.0	2.1	2.3	0	7.7	0.0

## Appendix A Calculation details

The details of the calculations comparing detectors are given here. First consider the calculations for Fig. 4 ▸. Formulas for calculating the effective counting rate for the filter/slits with a non-energy-resolving detector are given by Stern & Heald (1979 ▸). However, these formulas did not include consideration of the intrinsic background from inelastic scattering. For the backgrounds considered in that paper this is not critical, but will become important at large values of the background to signal. To generate the new calculations we determine separately the signal and background, and apply the appropriate filter absorption to each. This requires an estimate of the total scattering rate which, as discussed in §2.2, was taken at 4 × 10^9^ for 18% of 4π and an *I*_0_ of 10^13^. The background had two components: the contributions above the filter edge consisting primarily of the elastic scatter, and the inelastic scatter below the filter edge energy. The below-the-edge scatter is a range of energies but was approximated as being attenuated by a single filter absorption coefficient. This approximation only becomes important at large values of the background where filters by themselves are not very useful. Thus, it did not seem worthwhile to attempt a better calculation. The attenuated signal and background determine the effective counting rate that can be optimized by varying the filter thickness and detector solid angle as described below. To perform the optimizations the calculations were set up in an EXCEL spreadsheet and the solver function was used to maximize the effective counting rate.

For the SSD curve in Fig. 4 ▸ the calculation is simpler. The background ratio *R* is reduced to include only the background that leaks into the Cu ROI. As discussed in §3.1, this was measured to be 1.6% for large dilutions. The reduced *R* is then used in equation (2).

Similarly the crystal case is also straightforward as described in §3.4. The signal count is reduced by the crystal efficiency and the background ratio is reduced to include only the intrinsic background that is within the crystal diffraction bandwidth.

When the solid angles are varied we need to consider the effect of the beam polarization and the escape probability of the fluorescence and scattering. Typically reducing the solid angle will reduce the scattering faster than the fluorescence as long as the detector is centered along the polarization direction.

For the fluorescence signal the escape probability was determined using the geometry in Fig. 8 ▸. From a thin layer *dx* we have the following signal:

where *I* is the incoming intensity and μ_f_(*E*) is the absorption coefficient of the fluorescent element. The fluorescence escape probability is

Combining these and integrating over *x* for a thick sample gives

For the scattering contribution, the calculation is the same except μ(*E*_f_) becomes μ(*E*) and we have to add the polarization factor to the escape probability,

These expressions were integrated numerically over a square (equal range of φ and χ) detector face to determine the change in the fluorescence and scattering as the solid angle is changed. To facilitate the optimization calculations these curves were parameterized with polynomial equations as shown in Fig. 9 ▸. This shows the scattering going to zero at small angles. In the real case it is difficult to completely eliminate the scattering even at χ = φ = 0°. The beam is not 100% polarized and other forms of parasitic scattering can enter the detector. Thus, the optimizations always assume a limit on the minimum solid angle.

When the combined filter and SSD case is considered, the filter and SSD calculations above are combined. First the filter is applied to determine the beam reaching the SSD and then the SSD parameters are applied to determine the effective counting rate. In this case the approximation of a fixed absorption coefficient for the inelastic scattering is quite good since we are only interested in the inelastic scattering within the detector ROI.
